# The impact of COVID‐19 workflow changes on radiation oncology incident reporting

**DOI:** 10.1002/acm2.13742

**Published:** 2022-08-06

**Authors:** Matthew E. Volpini, Katie Lekx‐Toniolo, Robert Mahon, Lesley Buckley

**Affiliations:** ^1^ Division of Radiation Oncology The Ottawa Hospital Ottawa ON Canada

**Keywords:** COVID‐19, incident learning system, quality assurance

## Abstract

**Background:**

The Ottawa Hospital's Radiation Oncology program maintains the Incident Learning System (ILS)—a quality assurance program that consists of report submissions of errors and near misses arising from all major domains of radiation. In March 2020, the department adopted workflow changes to optimize patient and provider safety during the COVID‐19 pandemic.

**Purpose:**

In this study, we analyzed the number and type of ILS submissions pre‐ and postpandemic precautions to assess the impact of COVID‐19‐related workflow changes.

**Methods:**

ILS data was collected over six one‐year time periods between March 2016 and March 2021. For all time periods, the number of ILS submissions were counted. Each ILS submission was analyzed for the specific treatment domain from which it arose and its root cause, explaining the impetus for the error or near miss.

**Results:**

Since the onset of COVID‐19‐related workflow changes, the total number of ILS submissions have reduced by approximately 25%. Similarly, there were 30% fewer ILS submissions per number of treatment courses compared to prepandemic data. There was also an increase in the proportion of “treatment planning” ILS submissions and a 50% reduction in the proportion of “decision to treat” ILS submissions compared to previous years. Root cause analysis revealed there were more incidents attributable to “poor, incomplete, or unclear documentation” during the pandemic year.

**Conclusions:**

COVID‐19 workflow changes were associated with fewer ILS submissions, but a relative increase in submissions stemming from poor documentation and communication. It is imperative to analyze ILS submission data, particularly in a changing work environment, as it highlights the potential and realized mistakes that impact patient and staff safety.

## 1 INTRODUCTION

Radiotherapy departments are tasked with carrying out complex and potentially hazardous tasks in a safe and accurate manner. Within the radiation oncology environment, incident learning systems (ILS) form an integral part of a comprehensive quality and safety program.^1^, ^2^, ^3^, ^4^ Studies have shown that error rates are significantly reduced, and patient safety is improved through the use of an ILS.^4^, ^5^, ^6^

The Ottawa Hospital's Radiation Oncology program maintains an ILS as one part of a quality assurance program that ensures safe and effective radiotherapy treatment.^1^ All members of the radiation medicine team are encouraged to submit reports of errors and near misses arising from all major domains of radiation medicine including treatment simulation, planning, delivery, and patient care. Incidents are submitted to and logged in an electronic ILS, which has been specifically tailored for radiation oncology. The system has been in use since 2009. Incidents are investigated, categorized, and discussed by a multidisciplinary committee made up of medical physicists, physicians, dosimetrists, nurses, radiation therapists, and administrative personnel. The committee recommends and tracks corrective actions, including escalation of incidents, where appropriate. The system also reports relevant incidents to the national incident reporting system.^2^

A successful quality assurance program should promote a culture of learning and continuous improvement.^4^ The success of an ILS within that context requires that individuals recognize the importance of the system and feel comfortable reporting errors and near misses. This relies on the system being designed such that investigations are conducted in a nonpunitive way, using a just‐culture approach.^7^, ^8^ One indicator of a strong safety culture is the number of incidents submitted to the ILS, with a higher number showing more individuals engaged and willing to bring forward potential events.^9^, ^10^

In March 2020, the COVID‐19 pandemic forced rapid changes to healthcare workflows throughout the world. Early evidence shows that in addition to the direct impact of COVID‐19 on patients and staff, the increased workload and changing work environment created a more stressful work environment.^11^ Rapidly changing directives, high stress environments, and fatigue are all factors that can contribute to an increased risk of error.^12^, ^13^ As part of the hospital‐wide COVID‐19 preparedness plan, the Radiation Oncology Department adopted workflow changes to reduce the number of personnel required to be in hospital to perform their duties. For physicians, this meant a drastically reduced number of in‐person patient assessments, even for new consults. For other program staff, including physicists and dosimetrists, many carried out their duties remotely, away from the hospital. Many employees found themselves working in a new, high‐stress environment with new methods of communications and less face‐to‐face interaction with their colleagues. With unprecedented changes to work flow and working environment, our group sought to analyze the number and nature of reports submitted to the ILS for review.

## 2 METHODS

ILS data was collected and analyzed from six 12‐month time periods: 13 March to the following 12 March for 2020–2021 and 2021–2022, representing the 24 months following the onset of the local COVID‐19 restrictions and the four 12‐month periods preceding March 2020 (13 March to 12 March 2016–2017, 2017–2018, 2018–2019, and 2019–2020. For each time period, the total number of incidents, the number of new treatment courses, and number of treatment fractions were gathered. Patients undergoing a course of radiotherapy may be treated with a single fraction or as many as 35 fractions based on numerous factors including the type of cancer and the treatment intent. The number of new treatment courses assigned impacts the workload prior to the treatment delivery and includes the decision to treat, treatment planning, and booking stages. Conversely, the number of radiotherapy treatment fractions impacts the workload experienced on the linear accelerator treatment units. For this reason, data on both treatment courses and treatment fractions were collected.

For each ILS submission, the incident type, origin domain, and root causes were extracted from the database. Each of these parameters had been previously assigned at the time of the incident investigation. The incident type is classified as either actual or potential. An actual incident is when an unwanted or unexpected change from a normal system behavior causes adverse effect to the patient or equipment. A potential incident or “near miss” is an event that could have resulted in harm to a patient but did not, either by chance or through timely intervention. The origin domain represents the specific treatment domain from which the incident arose and is categorized as one of: decision to treat, treatment simulation, treatment planning, treatment delivery, quality assurance, or patient discharge. Each incident had also previously been assigned a root cause, selected from a pre‐defined list of causes built into the ILS framework. The collected data was analyzed for the number and type of ILS submissions and compared across the time periods.

## 3 RESULTS

### 3.1 Total and normalized number of incidents

**FIGURE 1:**
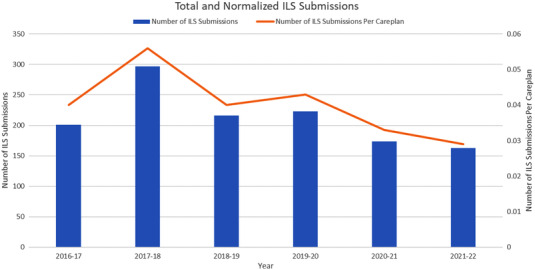
Total number of ILS submissions, and normalized number of ILS submissions per plan of care. During the pandemic years (2020–2021, 2021–2022), there were fewer total ILS submissions, and fewer ILS submissions per plan of care. Note “2018–2019” denotes 13 March 2018 to 12 March 2019, “2019–2020” denotes 13 March 2019 to 12 March 2020, and so forth

Incident totals and normalized data calculated against the total number of fractions are represented in Figure 1. In addition, the number of treatment plans for each time period can be found in Table 1. Submission data from the four most recent prepandemic years (2016–2017, 2017–2018, 2018–2019, and 2019–2020) showed an average of 234 annual ILS submissions, standard deviation (SD) 42.8.

**TABLE 1:** ILS submission data

Year	# of ILS submissions	# of treatment courses initiated	# of ILS submissions per treatment course	# of treatment fractions	Domain representation
2021–2022	163	5600	0.029	61,599	Treatment planning (27%) Treatment simulation (26%) Decision to treat (19%) Treatment delivery (17%) Quality assurance (12%)
2020–2021	174	5235	0.033	59,542	Treatment planning (28%) Treatment simulation (24%) Treatment delivery (20%) Decision to treat (16%) Quality assurance (13%)
2019–2020	223	5182	0.043	69,645	Decision to treat (34%) Treatment simulation (29%) Treatment planning (13%) Treatment delivery (13%) Quality assurance (11%)
2018–2019	216	5367	0.04	72,598	Decision to treat (32%) Treatment simulation (21%) Treatment planning (17%) Treatment delivery (17%) Quality assurance (12%)
2017–2018	297	5271	0.056	73,326	Decision to treat (46%) Treatment planning (16%) Treatment delivery (16%) Treatment simulation (14%) Quality assurance (7%)
2016–2017	201	4979	0.04	72,040	Decision to treat (30%) Treatment simulation (24%) Treatment planning (18%) Quality assurance (14%) Treatment delivery (13%)

*Note*: Normalized data is represented as the number of ILS submissions per treatment course initiated, as the majority of the ILS submissions originated from the decision to treat and planning stages of treatment, therefore not related to the total number of fractions

### 3.2 Origin domain

**FIGURE 2:**
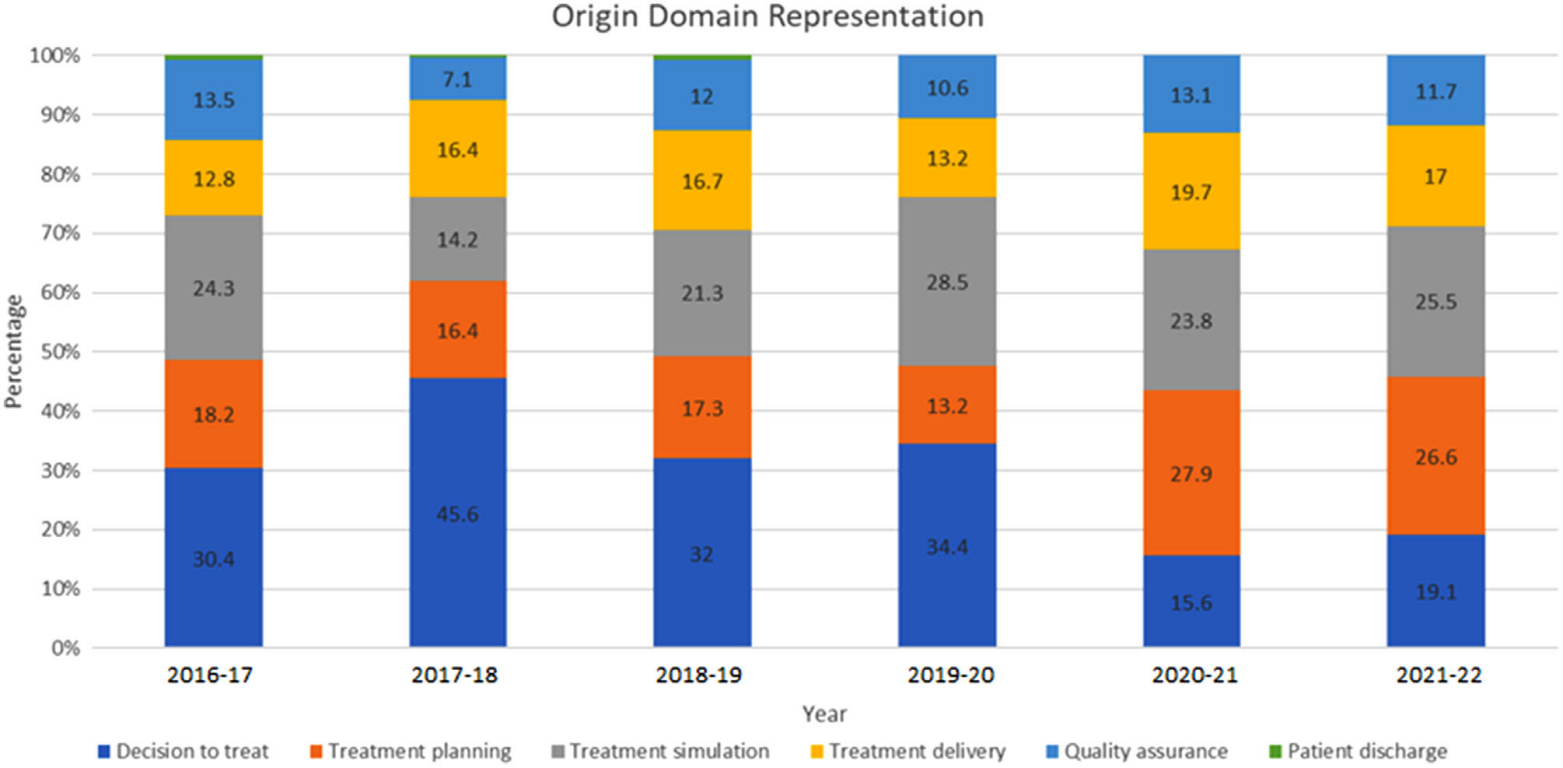
Normalized origin domain representation by year. During the pandemic years (2020–2021, 2021–2022), there was a relative decrease in the number of decision to treat ILS submissions, and relative increase in the number of treatment planning ILS submissions. Note “2018–2019” denotes 13 March 2018 to 12 March 2019, “2019–2020” denotes 13 March 2019 to 12 March 2020, and so forth

In 2016–2017, the most common ILS submission domains were decision to treat (30%), treatment simulation (24%), treatment planning (18%), and quality assurance (14%). In 2017–2018, the most common ILS submission domains were decision to treat (46%), treatment planning (16%), treatment delivery (16%), and treatment simulation (14%). In 2018–2019, the most common ILS submission domains were decision to treat (27%), treatment simulation (21%), treatment planning (17%), and treatment delivery (17%). The same top four domains were represented in 2019–2020 with decision to treat (34%), treatment simulation (29%), treatment planning (13%), and treatment delivery (13%). Based on the 4‐year average from the four prepandemic years (2016–2017, 2017–2018, 2018–2019, and 2019–2020), the most common domains represented were decision to treat (35.6%, standard deviation [SD] 6.9), treatment simulation (22.1%, SD 6%), treatment planning (16.3%, SD 2.2%), and treatment delivery (14.8%, SD 2.1%). Beginning in the 2020–2021 pandemic year, there was a shift in the relative distribution of origin domains with an increase in the number of treatment planning submissions and relatively few from the decision to treat domain. The most common four domains in 2020–2021 were treatment planning (28%) treatment simulation (24%), treatment delivery (20%), and decision to treat (15%). In 2021–2022, the most common domains were treatment planning (27%), treatment simulation (26%), decision to treat (19%), and treatment delivery (17%). In all years, the quality assurance domain was found to be one of the lowest represented domains with 14%, 7%, 12%, 11%, 13%, and 12% in 2016–2017, 2017–2018, 2018–2019, 2019–2020, 2020–2021, and 2021–2022, respectively. The patient discharge domain was also consistently low, with only a single case in the 2016–2017, 2017–2018, and 2018–2019 time periods and no cases reported in 2019–2020, 2020–2021, 2021–2022 years. This is summarized in Figure 2.

### 3.3 Categories and root causes

**FIGURE 3:**
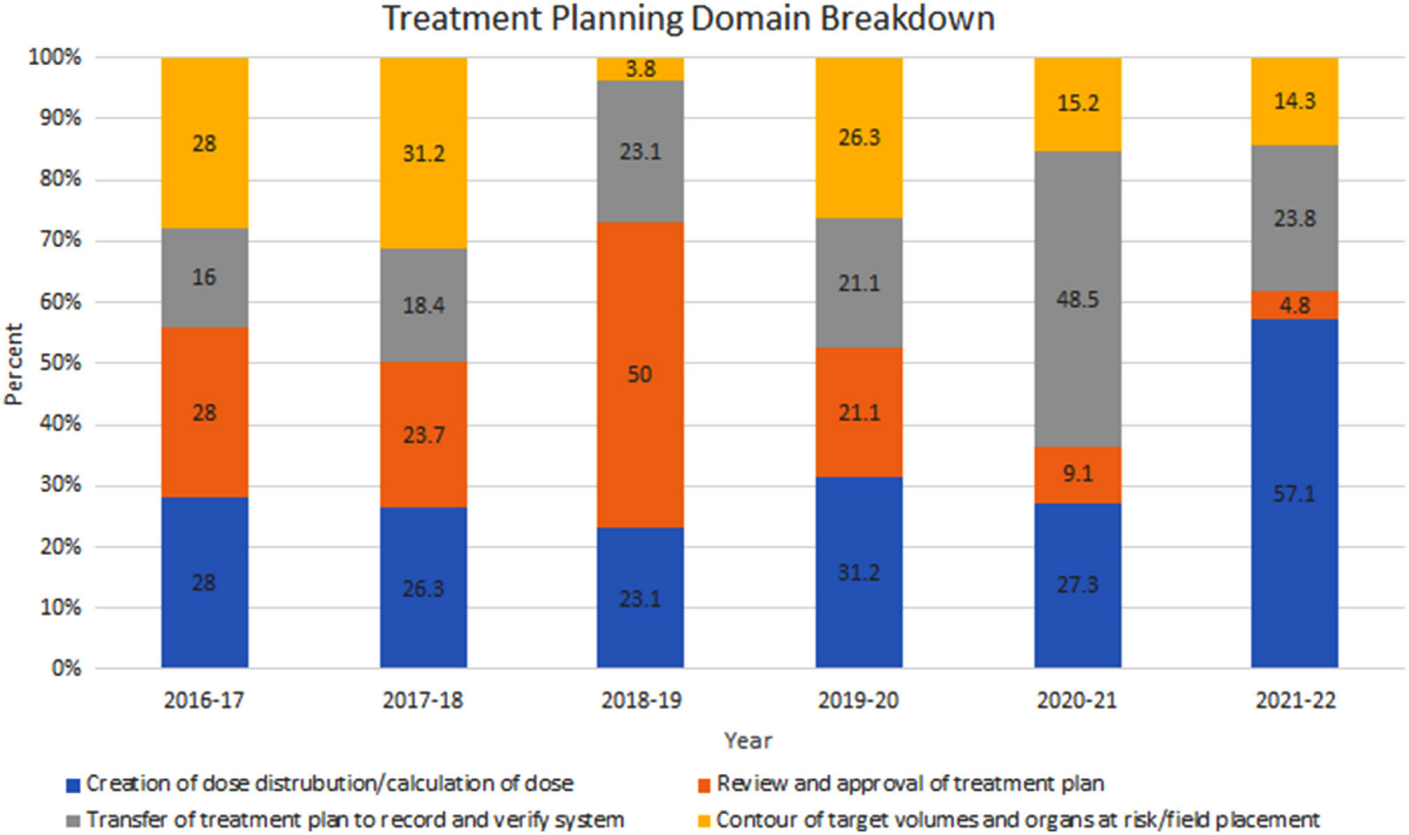
Normalized representation of the treatment planning domain categories. Note “2018–2019” denotes 13 March 2018 to 12 March 2019, “2019–2020” denotes 13 March 2019 to 12 March 2020, and so forth

As the number of ILS submissions originating from the treatment planning domain increased during the pandemic years, ILS submissions from this domain were further characterized. During the first pandemic year (2020–2021), 48.5% of treatment planning submissions were from the “transfer of treatment plan to record and verify” category compared with a prepandemic average of 19.7% (SD 3.1%). This category refers to the any part of the treatment plan preparation process where the plan documentation or treatment instructions from the treatment planning software are transferred into the record and verify system used to drive the patient treatment. Examples of these submissions include missing plan documents or approvals, incorrect shifts entered into the record and verify software, and incorrect setup fields. During the second pandemic year (2021–2022), 57.1% of treatment planning submissions were from the “creation of dose distribution and calculation of dose” category compared with a prepandemic average of 27.2% (SD 3.4%). An additional notable change during the pandemic years is the proportion of “review and approval of treatment plan” submissions. The prepandemic average for this category was 30.7% (SD 13.2%), while the pandemic values were 9.1% and 4.8% for the 2020–2021 and 2021–2022 pandemic years, respectively. There were no other notable differences in category representation across the years studied (Figure 3).

As the number of ILS submissions originating from the decision to treat domain were reduced during the pandemic years, ILS submissions from this domain were further characterized. On root cause analysis, there were no clear trends in the “policy not followed” cause, accounting for 29%, 12%, 21%, 15%, 25%, and 11% of all decision to treat—specific ILS submissions for the 6 years considered. “Policy not followed” could include any submission where the cause was determined to be a deviation from a policy already in place. Furthermore, the “poor, incomplete, unclear, missing documentation” root cause showed a relative increase in the first pandemic year as a percentage of all decision to treat ILS submissions. Within the “decision to treat” category, examples of incomplete documentation may include missing prescription information, poorly communicated changes to patient scheduling, or unclear instructions. The percentage of decision to treat ILS submissions due to poor or missing documentation for the 6 years starting in 2016–2017 and ending with the 2021–2022 year were 8.9%, 3.8%, 6.2%, 11.5%, 20%, and 5.6%. Finally, the “plan forgotten in progress” or “loss of attention” root causes accounted for 15% and 16% of the decision to treat—specific ILS submission for the two pandemic years 2020–2021 and 2021–2022, respectively, but only 6.7%, 4.8%, 4.2%, and 5.8% for the four preceding years. These root causes are assigned when the investigation shows that there were competing priorities or environmental distractions that led to the staff member being unable to devote sufficient focus to the task at hand.

## 4 DISCUSSION

### 4.1 Number of incidents

During the most recent 4 years prior to the onset of COVID‐19 workflow changes, 13 March 2016 to 12 March 2020, there were an average of 234 ILS submissions per 12‐month time period (SD 43). Following the implementation of COVID‐related workflow changes, there were 174 ILS submissions from 13 March 2020 to 12 March 2021, and 163 ILS submissions from 13 March 2021 to 12 March 2022. This represents an approximate 25% reduction in the number of ILS submissions from baseline since the onset of COVID‐19‐related precautions. Initially, it was postulated that these reduced submission numbers could be related to decreased patient caseload; however, normalized ILS submission data (the number of ILS submissions per number of treatment courses) revealed a 4‐year pre‐pandemic average of 0.045 (SD 0.008), while the normalized value for the 2020–2021 pandemic year was 0.033, and for the most recent 2021–2022 year was 0.029. Therefore, the normalized ILS values following the implementation of COVID‐19 workflow protocols were lower during the pandemic years, with an average rate of 0.031, almost 30% lower than prior years. Prior studies have shown that the number of ILS submissions are correlated with the level of staff engagement.^9^, ^10^ While it may be possible that pandemic workflow changes led to fewer ILS‐worthy events, this seems unlikely as changes to long‐established workflow norms are more commonly associated with an increase in near misses and potential events as team members become acclimated to their new work environment and modified work conditions.^10^, ^14^, ^15^ Therefore, it is possible that the decrease in ILS submissions is indicative of decreased engagement with ILS submissions as team members worked to balance their duties with rapidly changing hospital policy, patient care restrictions, and workflow changes.^16^ To address this potential reason for the decreased ILS usage, the ILS committee sought to increase staff engagement through increased communications and improved feedback with the hope of increasing submissions since engagement in the ILS is essential during times of significant workflow changes. Further monitoring of the ILS submissions will be required to determine if the rate of submissions returns to prepandemic levels.

While the number of radiotherapy treatment courses was similar when comparing prepandemic years to the two pandemic periods, the number of fractions delivered was much lower. The average number of fractions delivered over the 12‐month periods prepandemic was 71,902 (SD 1594), whereas for the two time periods considered following the onset of the pandemic, the average number of fractions over 12 months was 60,570. Comparable numbers of treatment courses but fewer fractions is reflective of the department attempt to employ hypofractionated radiotherapy regimens where feasible in order to limit pandemic exposures to both patients and staff. With fewer fractions delivered during the pandemic years, we might expect there to be a smaller proportion of ILS submissions originating from the treatment delivery domain. Interestingly, however, the proportion of treatment delivery ILS submissions was higher during the pandemic years—18.5% compared with an average of 14.7% (SD 2.1%) during the prepandemic years. During the pandemic, on‐treatment workflows were disrupted by time‐consuming infection control requirements (including PPE requirements for radiation therapists and patients alike) combined with limited numbers of radiation therapists. This amounts to increased treatment complexity, which has been shown to be associated with increased error rates and is one potential explanation for the observed increase in treatment planning submissions during the pandemic years.^14^, ^15^

### 4.2 Breakdown by origin domain

During the prepandemic years, the treatment planning domain accounted for an average of 16.3% of ILS submissions (SD 2.2%), while there was a comparative increase in treatment planning submissions during the pandemic years, 28% and 27% for the 2020–2021 and 2021–2022 years, respectively. On analysis of the specific category of treatment planning submissions, it was found that there were two noteworthy changes in submission patterns during the pandemic years. During the first pandemic year, 48.5% of treatment planning submissions were from the “transfer of treatment plan to record and verify” category, more than twice the prepandemic average. Transfer of treatment plan to record and verify ILS submissions often arise from events that require multiple team members (physicists, dosimetrists, and physicians) to coordinate treatment plan amendments. Amid pandemic precautions, team members had less face‐to‐face interaction, relying instead on electronic forms of communication, which may not be as effective. Increased workload and communication breakdown are known sources of error in the radiation oncology work flow.^14^, ^17^ In addition, our institution initiated manual entry of couch movements just before initiation of pandemic precautions. This workflow alteration may also account for the increase in the proportion of “transfer of treatment plan to record and verify” submissions during the first pandemic year. The observed increase then returned to baseline during the second pandemic year, as the workflow alteration was no longer novel, and the affected team members acclimated to the change. During the second pandemic year (2021–2022), 57.1% of treatment planning submissions were from the “creation of dose distribution and calculation of dose” category, again, more than twice the prepandemic average to determine the etiology.

Finally, for both pandemic years, the proportion of “review and approval of treatment plan” submissions was observed to be less than a quarter of the prepandemic average for this category. These ILS submissions typically arise from changes made to the treatment plan or prescription that were not clearly communicated or documented. Prior to these years, changes were made to the electronic workflow that automated more of the process when a change is made, which has led to a reduction in the number of submissions related to the review and approval of treatment plans. Therefore, it is thought that this change in relative frequency is due to intentional process changes rather than effects of pandemic‐related issues.

During the pandemic year, there were an average of 50% fewer decision to treat ILS submissions compared to previous years. Root cause analysis showed no difference in the proportion of “policy not followed” submissions, suggesting good compliance with rapidly changing hospital and radiation oncology‐specific workflow changes. Conversely, the relative number of “poor, incomplete, unclear, and missing documentation” decision to treat ILS reports more than doubled during the first pandemic year. With more people working from home, clear documentation becomes paramount to effective workflow. This relative increase may be reflective of a change in the mode of communication during the pandemic, with a greater reliance on electronic documentation, and fewer in‐person interactions. This relative increase was not seen during the second pandemic year, which could be the result of a greater number of staff returning to on‐site work so that communication patterns more closely resembled the prepandemic environment and it could also be indicative of the adjustments made by staff to the increased reliance on electronic forms of communication. Alternatively, the increase in the proportion of poor, incomplete, unclear, and missing documentation reports may represent poorer staff documentation under the weight of rapidly changing clinical and clerical duties during the first pandemic year. Within only 2 years of pandemic data, it is difficult to draw conclusions as to why there was a spike in the relative incidence of poor documentation as a root cause. Regardless of why the changes occurred, it has been well documented that poorer communication leads to a greater frequency of ILS events, and our data is consistent with this finding.^14^, ^15^

Decision to treat ILS submissions were significantly increased for the “plan forgotten in progress” or “loss of attention” root causes during the pandemic year—more than seven times that from preceding years, on average. With fewer team members working in person, a greater number of competing interests including personal and patient PPE, infection control precautions, and a change to the type of radiotherapy courses (greater number of hypofractionated courses), it is reasonable to posit that such factors contributed to lapses in maintaining focus on the intended radiotherapy plan.

Finally, the “quality assurance” domain consistently accounted for < 15% of ILS submissions across all time periods studied. This is understandable, as quality assurance processes are designed to detect ILS‐worthy events and are themselves less likely to account for a significant number of ILS submissions.

## 5 CONCLUSION

Safe and effective patient care is the goal of any radiation oncology program. During the pandemic years, workflow and staffing changes were associated with a decrease in the number of ILS submissions, indicating less engagement with a vital component of quality assurance and therefore patient safety. In addition, significant changes observed regarding the types of submissions reported during the pandemic years are reflective of the unique challenges encountered during pandemic precautions. Continued engagement with ILS reporting is essential to the continued safety of the radiation oncology program, particularly during the dynamic COVID‐19 pandemic or other periods of rapid change within a program. Incident learning committees should be aware of potential competing priorities during such times and actively work to ensure that the ILS continues to play a key role in promoting learning and quality improvements.

## CONFLICTS OF INTEREST

No conflict of interest.

## AUTHOR CONTRIBUTIONS

*Conceptualization*: Lesley Buckley, Katie Lekx‐Toniolo, Robert Mahon, Matthew E. Volpini. *Methodology*: Lesley Buckley, Katie Lekx‐Toniolo, Matthew E. Volpini; *Data collection*: Lesley Buckley, Katie Lekx‐Toniolo, Matthew E. Volpini. *Data analysis*: Lesley Buckley, Matthew E. Volpini. *Data interpretation*: Matthew E. Volpini, Lesley Buckley, Katie Lekx‐Toniolo, Robert Mahon. *Writing—original draft preparation*: Matthew E. Volpini, Lesley Buckley. *Writing—review and editing*: Lesley Buckley, Katie Lekx‐Toniolo, Robert Mahon. Supervision: Lesley Buckley.
